# A review of health behaviour theories: how useful are these for developing interventions to promote long-term medication adherence for TB and HIV/AIDS?

**DOI:** 10.1186/1471-2458-7-104

**Published:** 2007-06-11

**Authors:** Salla Munro, Simon Lewin, Tanya Swart, Jimmy Volmink

**Affiliations:** 1 South African Cochrane Centre, Medical Research Council of South Africa, P.O. Box 19070, Tygerberg 7505, Cape Town, South Africa; 2 Health Systems Research Unit, Medical Research Council of South Africa and Department of Public Health and Policy London School of Hygiene and Tropical Medicine, Keppel Street London WC1E7 HT, UK; 3 Department of Psychology School of Human & Community Development, University of the Witwatersrand Private Bag X3, Wits, 2050, South Africa; 4 South African Cochrane Centre, Medical Research Council of South Africa and Deputy Dean: Research Faculty of Health Sciences, Stellenbosch University PO Box 19063, Tygerberg 7505, Cape Town, South Africa

## Abstract

**Background:**

Suboptimal treatment adherence remains a barrier to the control of many infectious diseases, including tuberculosis and HIV/AIDS, which contribute significantly to the global disease burden. However, few of the many interventions developed to address this issue explicitly draw on theories of health behaviour. Such theories could contribute to the design of more effective interventions to promote treatment adherence and to improving assessments of the transferability of these interventions across different health issues and settings.

**Methods:**

This paper reviews behaviour change theories applicable to long-term treatment adherence; assesses the evidence for their effectiveness in predicting behaviour change; and examines the implications of these findings for developing strategies to improve TB and HIV/AIDS medication adherence. We searched a number of electronic databases for theories of behaviour change. Eleven theories were examined.

**Results:**

Little empirical evidence was located on the effectiveness of these theories in promoting adherence. However, several models have the potential to both improve understanding of adherence behaviours and contribute to the design of more effective interventions to promote adherence to TB and HIV/AIDS medication.

**Conclusion:**

Further research and analysis is needed urgently to determine which models might best improve adherence to long-term treatment regimens.

## Background

Theories may assist in the design of behaviour change interventions in various ways [1-3], by promoting an understanding of health behaviour, directing research and facilitating the transferability of an intervention from one health issue, geographical area or healthcare setting to another.

Ensuring treatment adherence presents a considerable challenge to health initiatives. Haynes et al. ([4], p2) have defined adherence as "the extent to which patients follow the instructions they are given for prescribed treatments". Adherence is a more neutral term than 'compliance', which can be construed as being judgmental. While programmes promoting adherence have focused on various health behaviours, this review focuses specifically on long-term adherence to tuberculosis (TB) and HIV/AIDS treatment. Non-adherence to treatment for these diseases has severe human, economic and social costs. Interrupted treatment may reduce treatment efficacy and cause drug resistance [5], resulting in increased morbidity and mortality and further infections. Without intervention, adherence rates to long-term medication in high income countries are approximately 50% [6], while adherence in low and middle income countries may be even lower [7].

TB and HIV present particular challenges to adherence. Both are chronic and infectious diseases that affect mainly the most disadvantaged populations and involve complex treatment regimens with potentially severe side effects; both are public health priorities and non-adherence may cause drug resistance [7]. These characteristics differentiate these diseases from other chronic diseases such as asthma and hypertension where, for example, drug resistance is not a key issue. Treatment adherence is also affected by beliefs about the origins, transmission and treatment of TB and HIV, often resulting in the stigmatisation of those affected [7]. The interaction of these factors make adherence for these diseases not only a priority but a complex health issue.

Various interventions have been designed to improve treatment adherence, but few theories describe specifically the processes involved. Currently, there are more than 30 psychological theories of behaviour change [8], making it difficult to choose the most appropriate one when designing interventions. This is a particular problem within the field of adherence to long-term medications, where the consequences of non-adherence may be severe. Existing theories therefore need to be examined further to determine their relevance to the issue of long-term medication adherence.

Leventhal and Cameron [9] identified five main theoretical perspectives related to adherence: 1) biomedical; 2) behavioural; 3) communication; 4) cognitive; and 5) self-regulatory. Each perspective encompasses several theories. More recently, the stage perspective has emerged, which includes the transtheoretical model. The most commonly used theories are those within the cognitive perspective [1,10] and the transtheoretical model [1]. This review includes a short description of theories within each of the five perspectives listed above, as well as the transtheoretical model. We locate these theories specifically within the realm of adherence to long-term medication, defined as medication regimens of three months or more; describe their key characteristics and evidence base; and examine their relevance and applicability with regard to adherence to long-term medication regimens for TB and HIV/AIDS. To our knowledge, the area of long-term adherence to medication has not yet been addressed in reviews of health behaviour theories.

While the focus of this review is on factors affecting consumers, we acknowledge that adherence is a complex and dynamic phenomenon, which relates to consumers, providers, health systems and broader socio-economic and political contexts. Although the theories chosen for this review focus mainly on providers and consumers, this is not the only area in which adherence can be promoted. The review is intended as an information source for those wishing to develop theory-based interventions focusing on intra- or interpersonal factors to increase TB and/or HIV treatment adherence.

## Methods

A search was performed on MEDLINE, CINAHL, Pre-CINAHL, PsycInfo, ScienceDirect and ERIC databases using the keywords 'health and behaviour and (model or theory)'; '(model or theory); (adherence or concordance or compliance)', from the start date of each database to February 2005. Additional searches were performed in the University of Cape Town library, Google and Google Scholar. Citations were also identified from included papers. Finally, all databases consulted were searched again using the names of theories as keywords, with 'meta-analysis' or 'systematic review' in April 2005. Experts were consulted for comments and references. Published articles or book chapters in English, describing a particular theory, and articles that presented a meta-analysis of the theory, were included. Articles were excluded if they did not satisfy the aforementioned criteria. Where possible, interventions related to TB or HIV adherence were identified. No authors were contacted. Several additional randomised controlled studies or other articles were also included as examples of the use of theories in intervention development. In this paper we use the term 'theory', instead of 'model', and the term 'variable', instead of 'construct', when referring to a part of the theory.

## Results

Table 1 presents the theories included in this article and references to meta-analyses synthesizing the evidence for each. Below, we summarise each perspective and the theories within it and provide examples of its application to adherence behaviours [see additional file 1]. We then examine the usefulness of these theories in developing interventions to promote long-term adherence.

**Table 1:** Summary of selected health behaviour theories*

**Model**	**Author**	**Meta-analyses examining the model**	**Evidence supporting theory**
Biomedical		None identified (NI)	
BLT	Skinner, 1953	NI	
Communication		NI	
HBM	Rosenstock et al. 1966	1. 30 2. 31	1. 46 studies- substantial empirical support. 2. 16 studies; at best 10% of variance accounted for by any one dimension of the theory.
SCT	Bandura 1950's	38	27 studies; self-efficacy explained between 4% and 26% of variance
TRA	Fishbein & Ajzen, 1975	41	Theory explains about 25% of variance in behaviour from intention alone, and explains slightly less than 50% of variance in intentions.
TPB	Fishbein & Ajzen, 1975	1.43 2. 44 3. 45	1. 13 studies; 75% of interventions effected a change in behaviour in desired direction. 2. 56 studies; About a third of the variations in behaviour can be explained by the combined effect of intention and perceived behavioural control in the domain of health. 3. 185 independent empirical tests: combined effect of intention and perceived behavioural control explained about a third of variation in behaviour. Theory can explain 20% of prospective measures of actual behaviour.
PMT	Rogers, 1975	35	65 studies – Moderate effects in predicting behaviour.
Self-regulation	Leventhal et al. 1980	NI	
IMB	Fisher and Fisher 1992	NI	
TTM	Prochaska & DiClemente 1983	1. 58 2. 59	1. Stage based interventions not more effective at increasing smoking cessation than non-stage based interventions. 2. 91 independent samples. Results support that individuals use all 10 processes of change.

* The studies included in most of these meta-analyses covered a wide range of content areas, most not directly related to adherence behaviour. Readers are encouraged to consult the original source for topic coverage.

### The biomedical perspective

The biomedical perspective incorporates the biomedical theory in which patients are assumed to be passive recipients of doctors' instructions [11]. Health or disease is traced back to biomedical causes, such as bacteria or viruses, and treatment is therefore focused on the patient's body [11]. In keeping with this mechanistic view of illness, mechanical solutions, such as prescribed pills, are preferred [12]; non-adherence is understood to be caused by patient characteristics, such as age and gender [12]. Technological innovations to promote adherence, such as Medication Event Monitoring Systems ^®^, are sometimes rooted in this perspective [7]. However, despite its implicit use by many health professionals, this perspective is infrequently used explicitly in interventions.

A fundamental limitation of this theory is that it ignores factors other than patient characteristics that may impact on health behaviours – for example, patients' perspectives of their own illness [7]; psycho-social influences [12]; and the impacts of the socio-economic environment. The socio-economic environment or demographics may, however, be markers for other factors that lend themselves to intervention even though they themselves cannot be changed [13]. The danger of using demographics as proxy variables for adherence is that certain groups that come to be seen as "lost causes" may be excluded (e.g. [14]). This biomedical theory has recently been integrated into a larger "biopsycho-socio-environmental" theory, which incorporates the wider socio-environmental context [11]. However, this theory is not located strictly within the biomedical approach. Due to the assumption that patients are passive and the focus on biomedical factors, it is unlikely that the biomedical theory can contribute significantly to TB or HIV medication adherence. Patients are generally *active *decision makers and do not merely receive and follow instructions passively. No meta-analyses specifically examining this perspective were identified.

### Behavioural (learning) perspective

This perspective incorporates behavioural learning theory (BLT) which is focused on the environment and the teaching of skills to manage adherence [7]. It is characterised by the use of the principles of antecedents and consequences and their influence on behaviour. Antecedents are either internal (thoughts) or external (environmental cues) while consequences may be punishments or rewards for a behaviour. The probability of a patient following a specific behaviour will partially depend on these variables [7].

Adherence promoting strategies informed by this perspective, such as patient reminders, have been found to improve adherence [15]. Several interventions incorporating elements of BLT have also been reported to be effective for adherence to long-term medications [4]. However, a more recent meta-analysis examining adherence to highly active antiretroviral (ARV) therapy concluded that interventions with cue dosing and external rewards – approaches derived from BLT -were as efficacious as those without [16]. Another randomised controlled trial on ARVs reported a negative effect when using electronic reminder systems [17]. Further evidence is therefore needed on the effectiveness of these types of strategy.

BLT has been critiqued for lacking an individualised approach and for not considering less conscious influences on behaviour not linked to immediate rewards [12]. These influences include, for example, past behaviour, habits, or lack of acceptance of a diagnosis. The theory is limited, too, by its focus on external influences on behaviour. Programme planners should therefore consider carefully individuals' perceptions of appropriate rewards before using such theory to inform programme design. Interventions drawing on behavioural theory are often used in combination with other approaches, although seldom explicitly. No meta-analyses were found that examined this perspective.

### Communication perspective

Communication is said to be "the cornerstone of every patient-practitioner relationship" [[11], p. 56]. This perspective suggests that improved provider-client communication will enhance adherence [7,11] and implies that this can be achieved through patient education and good health care worker communication skills – an approach based on the notion that communication needs to be clear and comprehensible to be effective. It also places emphasis on the timing of treatment, instruction and comprehension. An example of an intervention utilising this perspective is one that aims to improve client-provider interaction. Critiques of this perspective argue that it ignores attitudinal, motivational and interpersonal factors that may interfere with the reception of the message and the translation of knowledge into behaviour change [12].

A number of reviews have examined the effects of interventions including communication elements [18-21]. However, few of these have examined the effects of communication on health behaviours specifically. Two reviews focusing on interventions to improve provider-client communication showed that these can improve communication in consultations, patient satisfaction with care [18] as well as health outcomes [21]. However, these reviews also show limited and mixed evidence on the effects of such interventions on patient health care behaviours, such as adherence.

Communication components have been used within several adherence interventions but seldom explicitly or as the main component. Such interventions are unlikely to succeed in isolation in improving long-term adherence to medications because of the influence of external factors, such as the costs of accessing healthcare for treatment. Communication interventions are also typically restricted to provider-client interactions and additional social or financial support may thus be required.

### Cognitive perspective

The cognitive perspective includes theories such as the health belief model (HBM), social-cognitive theory (SCT), the theories of reasoned action (TRA) and planned behaviour (TPB) and the protection motivation theory (PMT). These theories focus on cognitive variables as part of behaviour change, and share the assumption that attitudes and beliefs [22], as well as expectations of future events and outcomes [23], are major determinants of health related behaviour. In the face of various alternatives, these theories propose, individuals will choose the action that will lead most likely to positive outcomes.

These theories have noticeable weaknesses, however: firstly, that non-voluntary factors can affect behaviour [23]; devoting time to conscious deliberation regarding a repeated choice also seems uneconomical [22]. Secondly, these theories do not adequately address the behavioural skills needed to ensure adherence [7]. Thirdly, these theories give little attention to the origin of beliefs and how these beliefs may influence other behaviours [24]. In addition, it has been argued that they ignore other factors that may impact on adherence behaviour, such as power relationships and social reputations [25], and the possibility that risk behaviour may involve more than one person [26]. It has also been suggested that they focus on a single threat and prevention behaviour and do not include possible additional threats competing for the individual's attention [24].

#### Health Belief Model

The HBM views health behaviour change as based on a rational appraisal of the balance between the barriers to and benefits of action [12]. According to this model, the perceived seriousness of, and susceptibility to, a disease influence individual's perceived threat of disease. Similarly, perceived benefits and perceived barriers influence perceptions of the effectiveness of health behaviour. In turn, demographic and socio-psychological variables influence both perceived susceptibility and perceived seriousness, and the perceived benefits and perceived barriers to action [1,7]. Perceived threat is influenced by cues to action, which can be internal (e.g. symptom perception) or external (e.g. health communication) (Rosenstock, 1974 in [7]).

High-perceived threat, low barriers and high perceived benefits to action increase the likelihood of engaging in the recommended behaviour [27]. Generally, all of the model's components are seen as independent predictors of health behaviour [28]. Bandura [29] notes, however, that perceived threats – especially perceived severity – have a weak correlation with health action and might even result in avoidance of protective action. Perceived severity may also not be as important as perceived susceptibility. Recently, self-efficacy was added into the theory [30], thereby incorporating the need to feel competent before effecting long-term change [31].

There are two main criticisms of this theory: firstly, the relationships between these variables have not been explicitly spelt out [32] and no definitions have been constructed for the individual components or clear rules of combination formulated [28]. It is assumed that the variables are not moderated by each other and have an additive effect [32]. If, for example, perceived seriousness is high and susceptibility is low, it is still assumed that the likelihood of action will be high -intuitively one might assume that the likelihood in this case would be lower than when both of the variables are high [22,32]. The HBM also assumes that variables affect health behaviour directly and remain unmoderated by behavioural intentions [22]. The second major weakness of HBM is that important determinants of health behaviour, such as the positive effects of negative behaviours and social influence, are not included [22,32]. In addition, some behaviours such as smoking are based on habits rather than decisions [33]. While the theory may predict adherence in some situations, it has not been found to do so for "risk reduction behaviours that are more linked to socially determined or unconscious motivations" [[12], p.165].

The two reviews identified that examined this theory had inconclusive results. A critical review [34] examined 19 studies which involved sick role behaviours, such as compliance to antihypertensive medication. While the four dimensions of the model produced significant effects in most of the studies included [34], the studies had considerable methodological gaps. A more recent meta-analysis [35] indicated that while the HBM was capable of predicting 10% of variance in behaviour at best, the included studies were heterogeneous and were unable to support conclusions as to the validity of the model. Therefore further studies are needed to assess the validity of this theory. When applying this theory to long-term medication adherence, it is also important for the influence of socio-psychological factors to be considered. For example, cultural beliefs about TB – such as its relationship with witchcraft [36] – may reduce an adherence intervention's effectiveness.

#### The protection-motivation theory

According to this theory, behaviour change may be achieved by appealing to an individual's fears. Three components of fear arousal are postulated: the magnitude of harm of a depicted event; the probability of that event's occurrence; and the efficacy of the protective response [37]. These, it is contended, combine multiplicatively to determine the intensity of protection motivation [22], resulting in activity occurring as a result of a desire to protect oneself from danger [37]. This is the only theory within the broader cognitive perspective that explicitly uses the costs and benefits of existing and recommended behaviour to predict the likelihood of change [23].

An important limitation of this theory is that not all environmental and cognitive variables that could impact on attitude change (such as the pressure to conform to social norms) are identified [37]. The most recent version of the theory assumes that the motivation to protect oneself from danger is a positive linear function of beliefs that: the threat is severe, one is personally vulnerable, one can perform the coping response (self efficacy) and the coping response is effective (response efficacy) [22]. Beliefs that health-impairing behaviour is rewarding but that giving it up is costly are assumed to have a negative effect [22]. However, the subdivision of perceived efficacy into categories of response and self efficacy is perhaps inappropriate – people would not consider themselves capable of performing an action without the means to do it [29].

A meta-analysis examining this theory found only moderate effects on behaviour [39]. The revised PMT may be less cumbersome to use than the TRA – it also does not assume that behaviour is always rational. [39]. The PMT may be appropriate for adherence interventions as it is unlikely that an individual consciously re-evaluates all of their routine behaviours such as, for example, taking long-term medication. However, the influence of social, psychological and environmental factors on motivation requires consideration by those using this approach.

#### Social-cognitive theory

This theory evolved from social learning theory and may be the most comprehensive theory of behaviour change developed thus far [1]. It posits a multifaceted causal structure in the regulation of human motivation, action and well-being [40] and offers both predictors of adherence and guidelines for its promotion [29]. The basic organising principle of behaviour change proposed by this theory is reciprocal determinism in which there is a continuous, dynamic interaction between the individual, the environment and behaviour [1].

Social-cognitive theory suggests that while knowledge of health risks and benefits are a prerequisite to change, additional self-influences are necessary for change to occur [41]. Beliefs regarding personal efficacy are among some of these influences, and these play a central role in change. Health behaviour is also affected by the expected outcomes – which may be the positive and negative effects of the behaviour or the material losses and benefits. Outcomes may also be social, including social approval or disapproval of an action. A person's positive and negative self-evaluations of their health behaviour and health status may also influence the outcome. Other determinants of behaviour are perceived facilitators and barriers. Behaviour change may be due to the reduction or elimination of barriers [41]. In sum, this theory proposes that behaviours are enacted if people perceive that they have control over the outcome, that there are few external barriers and when individuals have confidence in their ability to execute the behaviour [28].

A review reported that self efficacy could explain between 4% and 26% of variance in behaviour [42]. However, this analysis was limited to studies of exercise behaviour, and did not include reports that examined SCT as a whole. Due to its wide-ranging focus, this theory is difficult to operationalise and is often used only in part [43], thus raising questions regarding its applicability to intervention development.

#### Theory of planned behaviour and the theory of reasoned action

The first work in this area was on the TRA [44].

The TRA assumes that most socially relevant behaviours are under volitional control, and that a person's intention to perform a particular behaviour is both the immediate determinant and the single best predictor of that behaviour [45]. An intention to perform a behaviour is influenced by attitudes towards the action, including the individual's positive or negative beliefs and evaluations of the outcome of the behaviour. It is also influenced by subjective norms, including the perceived expectations of important others (e.g. family or work colleagues) with regard to a person's behaviour; and the motivation for a person to comply with others' wishes. Behavioural intention, it is contended, then results in action [44]. The authors argue that other variables besides those described above can only influence the behaviour if such variables influence attitudes or subjective norms. A meta-analysis examining this theory found that it could explain approximately 25% of variance in behaviour in intention alone, and slightly less than 50% of variance in intentions [45]. This suggests that support for this theory is limited.

Additionally, The TRA omits the fact that behaviour may not always be under volitional control and the impacts of past behaviour on current behaviours [22]. Recognising this, the authors extended the theory to include behavioural control and termed this the TPB. 'Behavioural control' represents the perceived ease or difficulty of performing the behaviour and is a function of control beliefs [45]. Conceptually it is very similar to self-efficacy [22] and includes knowledge of relevant skills, experience, emotions, past track record and external circumstances (Ajzen, in [46]). Behavioural control is assumed to have a direct influence on intention [45]. Meta-analyses examining the TPB have found varied results regarding the effectiveness of the theory's components [47-49]. Although not conclusive, the results of the analyses are promising.

Sutton [45] suggests that the TRA and TPB require more conceptualisation, definition and additional explanatory factors. Attitudes and intentions can also be influenced by a variety of factors that are not outlined in the above theories [22]. Specifically, these theories are largely dependent on rational processes [50] and do not allow explicitly for the impacts of emotions or religious beliefs on behaviour, which may be relevant to stigmatised diseases like TB and HIV/AIDS.

#### Information-motivation-behavioural skills (IMB) theory

This theory was developed to promote contraceptive use and prevent HIV transmission. IMB was constructed to be conceptually based, generalisable and simple [51]. It has since been tailored specifically to designing interventions to promote adherence to ART [52]

This theory focuses on three components that result in behaviour change: information, motivation and behaviour skills. Information relates to the basic knowledge about a medical condition, and is an essential prerequisite for behaviour change but not necessarily sufficient in isolation [51]. A favourable intervention would establish the baseline levels of information, and target information gaps [51]. The second component, motivation, results from personal attitudes towards adherence; perceived social support for the behaviour; and the patients' subjective norm or perception of how others with the condition might behave [7]. Finally, behavioural skills include factors such as ensuring that the patient has the skills, tools and strategies to perform the behaviour as well as a sense of self-efficacy – the belief that they can achieve the behaviour [51].

The components mentioned above need to be directly relevant to the desired behaviour to be effective [7]. They can also be moderated by a range of contextual factors such as living conditions and access to health services [52]. Information and motivation are thought to activate behavioural skills, which in turn result in risk reduction behavioural change and maintenance [51]. The theory is said to be moderately effective in promoting behaviour change [7], and has been shown to have predictive value for ART adherence [53]. However, no meta-analyses were identified that assessed the effects of this model. The advantage of IMB is its simplicity and its recent application to ART adherence suggests that it may be a promising model for promoting adherence to TB medication.

### Self-regulation perspectives

Self-regulatory theory is the main theory in this domain. Developed to conceptualise the adherence process in a way that re-focuses on the patient [54], the theory proposes that it is necessary to examine individuals' subjective experience of health threats to understand the way in which they adapt to these threats. According to this theory, individuals form cognitive representations of health threats (and related emotional responses) that combine new information with past experiences [55]. These representations 'guide' their selection of particular strategies for coping with health threats, and consequently influence associated outcomes [56]. The theory is based on the assumption that people are motivated to avoid and treat illness threats and that people are active, self-regulating problem solvers [57]. Individuals, it is implicitly assumed, will endeavour to reach a state of internal equilibrium through testing coping strategies. The process of creating health threat representations and choosing coping strategies is assumed to be dynamic and informed by an individual's personality, and religious, social and cultural context [55]. In addition, a complex interplay exists between environmental perceptions, symptoms and beliefs about disease causation [54].

The self-regulation theory offers little guidance related to the design of interventions [7] and no meta-analyses examining evidence for the effectiveness of this theory were identified. While the theory seems intuitively appropriate, specific suggestions are needed as to how these processes could promote adherence.

### Stage perspectives

#### The transtheoretical model (TTM)

This theory is most prominent among the stage perspectives. It hypothesizes a number of qualitatively different, discrete stages and processes of change, and reasons that people move through these stages, typically relapsing and revisiting earlier stages before success [58,59]. This theory is said to offer an "integrative perspective on the structure of intentional change" [[60], p. 1102] – the perceived advantages and disadvantages of behaviour are crucial to behaviour change [61].

The process of change includes independent variables that assess how people change their behaviour [62] and the covert and overt activities that help individuals towards healthier behaviour [63]. Different processes are emphasised at different stages.

Criticisms of TTM include the stages postulated and their coverage and definitions, and descriptors of change. According to Bandura [40], this theory violates all three of the basic assumptions of stage theories: qualitative transformations across discrete stages, invariant sequence of change, and non-reversibility. In addition, the proposed stages may only be different points on a larger continuum [29,58,63]. Bandura [29] suggests that human functioning is too multifaceted to fit into separate, discrete stages and argues that stage thinking could constrain the scope of change-promoting interventions. Furthermore, TTM provides little information on *how *people change and *why *only some individuals succeed [28].

Sutton [56] argues that the stage definitions included in the TTM are logically flawed, and that the time periods assigned to each stage are arbitrary. Similarly, there is also a need for more attention to measurement, testing issues and definition of variables and causal relationships [58]. The coverage and type of processes included may also be inadequate [63].

The TTM has received much practitioner support over the years, but less direct research support for its efficacy [3,10]. The meta-analyses identified for this review did not offer direct support for the theory; while one found that individuals use all 10 processes of change [64], another found that interventions that used the stage perspective were not more efficient than those not using the theory [65]. Further evidence of its efficacy is therefore needed. A strength of this theory is that it allows interventions to be tailored to individual needs. However, large-scale implementation of these interventions may be time consuming, complicated and costly. Its use may be more appropriate in areas where rapid behaviour change is not necessary.

## Discussion

This review has discussed a number of health behaviour theories that contribute to understanding adherence to long-term medications, such as those for TB and HIV/AIDS.

Although the use of theory to develop interventions to promote adherence offers several advantages, it also has some limitations. Firstly, there is little evidence that allows for the direct comparison of these theories [66]. Combining studies based on even one theory, in order to perform a meta-analysis to assess its effectiveness in predicting behaviours, is difficult due to various methodological problems in the original studies [60]. Furthermore, the number of theories in this field has proliferated over time, as theorists have examined different areas of behaviour and engaged in re-examining existing explanatory theories. Researchers, health planners and practitioners may therefore be overwhelmed by the multitude of theories available to them and the fragmented, and often contradictory, evidence. Questions also remain regarding the applicability of these theories to contexts other than those in which they were developed. Ashing-Giwa [67], for example, suggests that the above theories do not address socio-cultural aspects sufficiently. Issues such as the stigma attached to TB due to its perceived relation to HIV (especially in developing countries) may impact on the acceptability and the uptake of interventions. Further attention should therefore be given to the question of whether theories developed in the USA and the UK are applicable to individuals in other contexts where the disease burden from HIV/AIDS and TB is greatest.

Secondly, health behaviour change theories have tended to encompass a wide variety of health behaviours, each qualitatively different. The systematic reviews identified for this paper included studies ranging from smoking cessation to mothers limiting babies' sugar intake. Particular theories may be more applicable than others to improving adherence to specific health behaviours. For example, adherence to long-term medication will necessarily be different to a behaviour change required to take up exercise. In addition, achieving adherence to TB medication may be seen as an urgent issue for public health because of its infectiousness, and the recent emergence of extremely drug resistant strains [68]. It is difficult therefore to compare the effects of the theories across health categories or even within individual categories.

Thirdly, few studies were identified that had examined the selected health behaviour theories in relation to long-term medication adherence, or that had developed interventions to promote long-term adherence explicitly based on these theories, particularly for TB. Sumartojo's [13] assessment that a theory-based approach has largely been absent within the field of TB behavioural research appears to remain valid today.

The application of theories to the design of interventions remains a challenge for researchers and programme planners [69] and there is considerable debate concerning the effectiveness and usefulness of theory in informing intervention development (see [2,70,71]). Despite a variety of studies in a variety of fields, or perhaps because of this variation, we would argue that there is no clear evidence yet for the support of any of these theories within the field of adherence behaviours. This is not to say that these theories cannot be useful – rather, we have insufficient evidence to conclusively determine this.

While these discussions continue, research should aim to shed light on the key questions related to the theory-intervention debate: Do sound theories result in effective interventions? Does an effective intervention constitute proof of a theory's value? How might theory be used to inform the design of an effective intervention? And how can a theory be reliably tested? Some research work has already been undertaken in these areas: in a systematic review of antiretroviral treatment adherence interventions, Amico et al. [72] found that the use of theory in constructing an intervention did not account for variability in the intervention's efficacy. However, it is unclear how many of the 24 included studies in this review articulated a health behaviour change theory or the extent to which this was done.

Two possible approaches have been suggested to addressing the difficulties raised by the multitude of existing theories on health behaviour change. One approach is to attempt to identify variables common to these theories. This has been undertaken for 33 health behaviour change theories [7] in order to make psychological theories more accessible and easier to select. The results of this study provide some guidance on the most important variables in psychological theories, and may assist in the further development of health behaviour change theories. A second approach is to attempt to integrate the theories. While there is a need for such theoretical integration [73], we argue that researchers and theorists alike should be cautious when picking and choosing parts of other theories to develop further theories – so-called "cafeteria-style theorizing" – as the resulting theories may include redundant variables [[29], p. 285].

Because some theories share overlapping variables describing using different names [8,41], and most differences are due to an emphasis of one variable over another [1], it would serve the development of this field to conduct studies to identify particular variables that perform best in predicting behaviour change. For example, in a meta-analysis of randomised controlled trials testing antiretroviral treatment adherence interventions, Simoni [16] found that giving basic information to patients, and engaging them in discussion about helping them to overcome cognitive factors, lack of motivation and unrealistic expectations about adherence, were effective in improving adherence. Similarly, comparative studies between theories could be used to identify effective components [74]. The field of health behaviour theory remains dynamic, and it is important to continue developing existing theories and approaches as new evidence emerges.

### Applying health behaviour theories to medication adherence for TB and HIV/AIDS

How optimal adherence for TB and HIV/AIDS can be ensured remains an important question. While large numbers of studies have explored patients' and health care providers' views regarding adherence to TB treatment [75] or have described programmes to improve adherence to these medications, there are still relatively few rigorous evaluations of interventions to promote adherence to TB and HIV/AIDS treatments [76,77]; even fewer have explicitly utilised behaviour change theories. For example, a systematic review of interventions to promote adherence to TB treatment [77] included ten trials, none of which used an explicit theoretical framework. A similar review identified seven different randomised controlled trials of interventions to promote adherence to antiretroviral therapy [76], of which only one employed an explicit theoretical framework. Similar figures have been reported in other domains: a review of guideline implementation studies showed that less than 10% of these provided an explicit theoretical rationale for their intervention [78]. Given the paucity of evidence to support any particular health behaviour theory, we cannot therefore suggest that these theories be used routinely to design adherence promoting interventions. However, since these theories may well have practical behaviour change potential, and since the problem of medication adherence remains significant for both clinical medicine and public health, further exploratory and explanatory research is needed.

A number of recommendations emerge from this review (Table 2): firstly, future research should focus not on the development of new theories but rather on the further examination of those already elaborated. Several key attributes that should be encompassed by theories explaining behaviour change have been suggested, including demonstrated effectiveness in predicting and explaining changes in behaviour across a range of domains; an ability to explain behaviour using modifiable factors; and an ability to generate clear, testable hypotheses. The theories should include non-volitional components (i.e. issues over which individuals do not have complete control) and take into account the influence of external factors, as perceived by individuals [2,70].

**Table 2:** Recommendations for using health behaviour theories to develop long-term adherence promoting interventions in TB and HIV

▪ Future research should focus on the further examination of existing theories.
▪ Further work is required to identify and explore health behaviour theories most applicable to improving adherence to long-term medications.
▪ Existing health behaviour models should be tested systematically.
▪ Interventions utilising health behaviour theories appropriately need to be developed and trialled.
▪ Reports of interventions to promote adherence to long-term medications for other health issues should be reviewed to explore how these have drawn on health behaviour theories.

Secondly, further work is required to identify theories of health behaviour that are most applicable to improving adherence to long-term medication. Existing health behaviour theories should be tested systematically to establish which best predict effects on different kinds of behaviour for different groups of people in different contexts. For example, does a particular theory predict changes in adherence behaviour for both men and women with TB in both England and South Africa? Some researchers have argued that experimental research and increased clarity in theories and methods could assist in the identification of effective behaviour change techniques, thereby contributing to the development of evidence-based practice in health psychology and implementation research [2,3]. Similar efforts need to be made regarding the use of theories as applied to adherence behaviour.

Thirdly, the abundance of theories and their poor evidence base highlights the need to develop and trial interventions that utilise these theories appropriately (i.e. in concordance with the theory), with well defined and operationalised variables. This will help to advance the study of human adherence behaviour and allow for better informed decisions related to how to these theories could be more widely applied in practice. (See references [2] and [75] for guidance on developing theoretically informed interventions). We have compiled a number of examples [see additional file 1] of the application of such theories in practice.

Finally, reports of interventions to promote adherence to long-term medications for other health issues, such as diabetes, asthma and hypertension, should be reviewed to determine how many have drawn on theory in the design and testing of these interventions; the range of theories utilised and the ways in which this was done; and the ways in which the use of theory contributed to understanding the effects of these interventions. Many reviews of such interventions exist (for example, see [83,84]) and these could act as a starting point for such work.

It is also important to list some of the limitations of this review. Firstly, we have been unable to capture all the available data on tests of health behaviour theories. Secondly, this paper examines only theories constructed by researchers and does not explore the health theories held by those receiving treatment. These lay theories of adherence with regard to antiretroviral [81] and TB treatment [75] are discussed elsewhere.

It should also be noted that any understanding of individual health behaviour, and interventions to change this, must be located within the relevant social, psychological, economic and physical environments [28]. Much research on adherence to TB medication has indicated that poor adherence is commonly the result of factors outside the individual's control, including clinic and health care organisation factors (such as interruptions to drug supply and long distances to health facilities) and structural factors (such as poverty and migration) [13,82,83]. Similar issues have been reported for adherence to ART [84]. Any focus on changing the behaviours of individuals with TB or HIV should not result in the neglect of these other dimensions or the further disadvantaging of the poor and vulnerable, thereby widening health disparities. Interventions that focus on providers, the provider-patient relationship, health system and contextual factors therefore also need to be developed and evaluated [76].

## Conclusion

There is no simple solution to the problem of adherence, or to the area of behaviour change. Health behaviour theories may shed light on the processes underlying behaviour change. However, an explicit theoretical basis is not always necessary for a successful intervention and further examination is needed to determine whether theory-based interventions in health care are more effective than those without an explicit theoretical foundation [2,70]. This review contributes to advancing this field by describing the commonly cited health behaviour theories, presenting the evidence and critique for each; discussing the applicability of these theories to adherence behaviour; and highlighting several recommendations for research and theory development. To understand and overcome the barriers to treatment adherence, considerable research is needed. However, given the importance of long-term medication adherence to global public health, particularly in relation to the HIV and TB epidemics, such research should receive much higher priority.

## Abbreviations

HIV/AIDS: Human immunodeficiency virus/Acquired immunodeficiency syndrome

TB: Tuberculosis

ARV: Antiretroviral

SCT: Social-cognitive theory

TRA: Theory of reasoned action

PMT: Protection motivation theory

HBM: Health belief model

TPB: Theory of planned behaviour

IMB: Information-motivation-behavioural skills model

ART: Antiretroviral therapy

TTM: Transtheoretical model

## Competing interests

The author(s) declare that they have no competing interests.

## Authors' contributions

JV and SL developed the idea for this paper, SM performed all searches and compiled the text, SL contributed to the writing and SL, TM and JV provided conceptual and editorial input. All authors read and approved the final manuscript.

**Figure 1:**
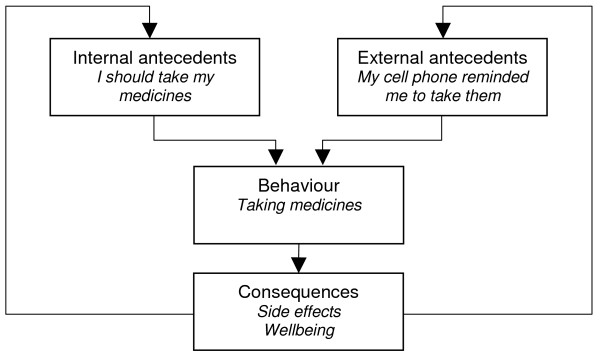
Behavioural learning theory.

**Figure 2:**
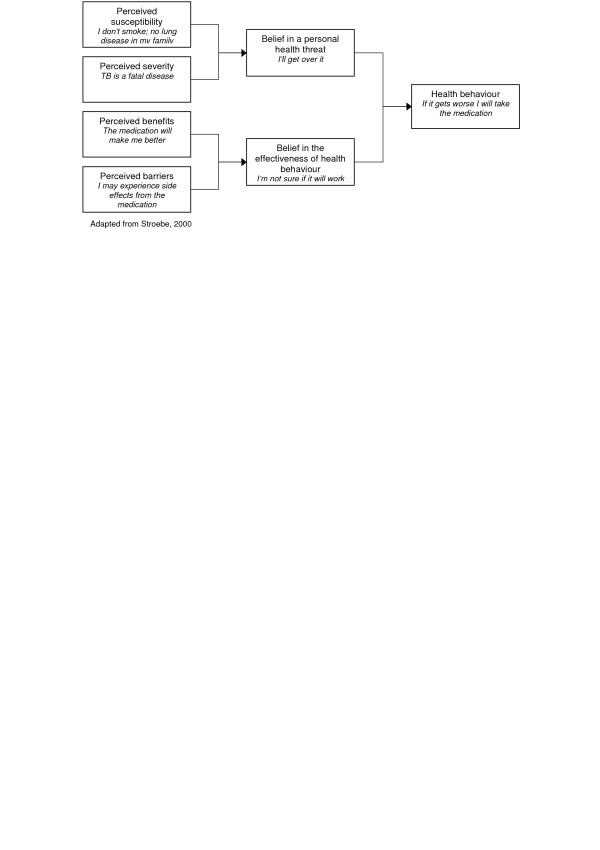
Health belief model.

**Figure 3:**
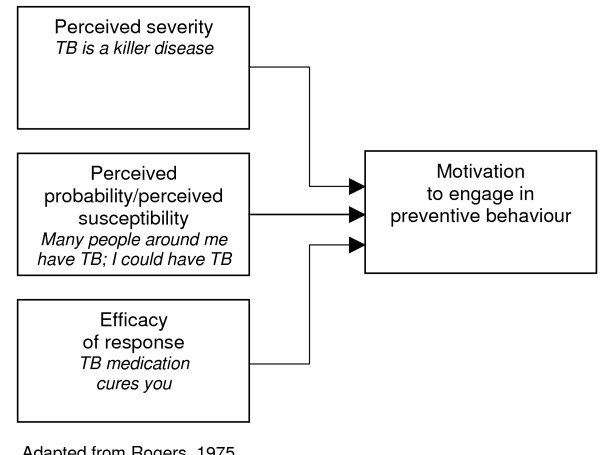
Protection motivation theory.

**Figure 4:**
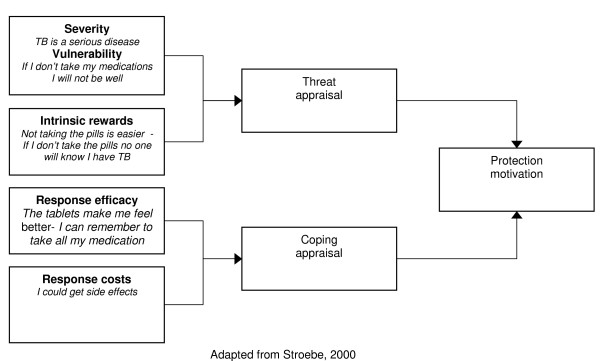
Revised protection motivation theory.

**Figure 5:**
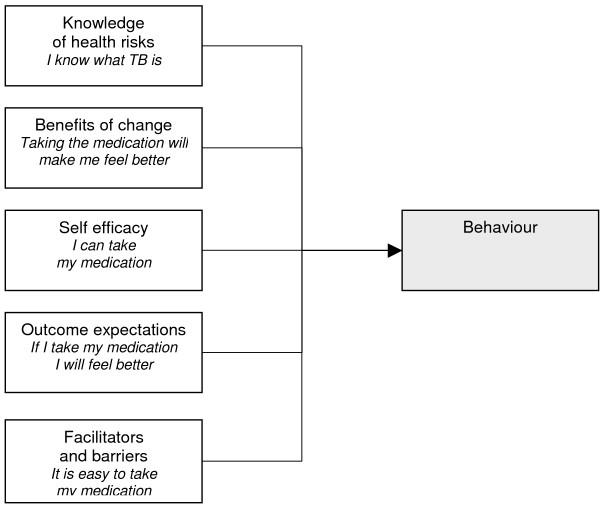
Social cognitive theory.

**Figure 6:**
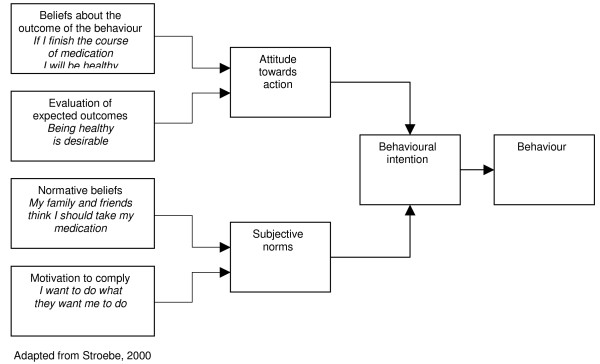
Theory of reasoned action.

**Figure 7:**
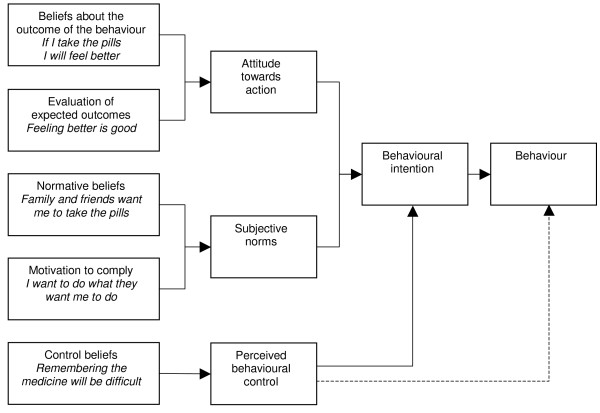
Theory of planned behaviour.

**Figure 8:**
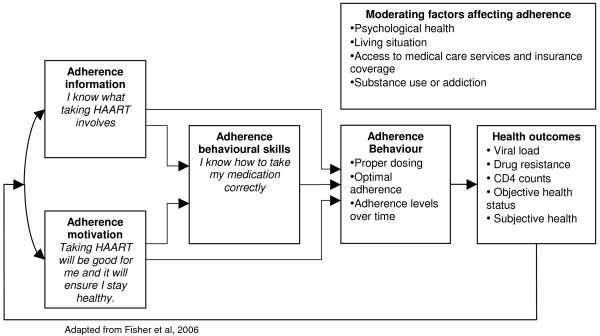
Information motivation behavioural skills model.

**Figure 9:**
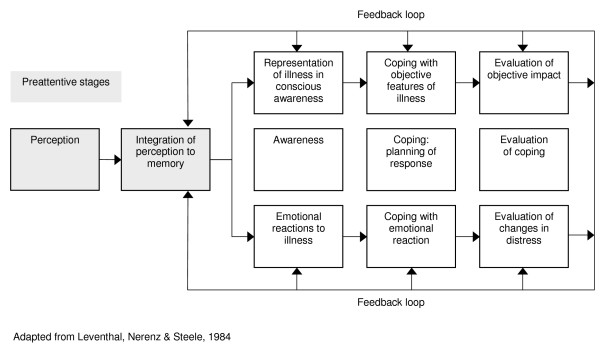
Self regulation theory.

**Figure 10:**
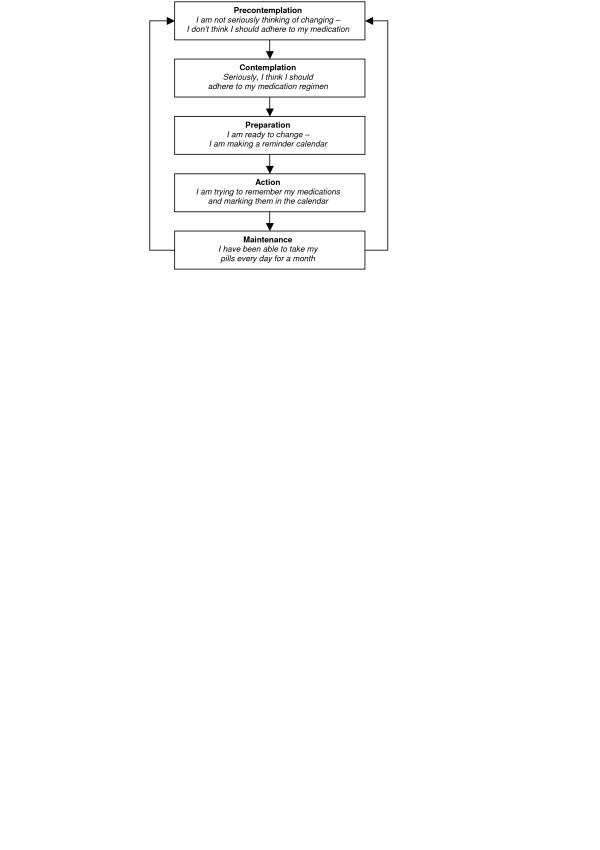
Transtheoretical model.

## Pre-publication history

The pre-publication history for this paper can be accessed here:



## Supplementary Material

Additional file 1 Examples of interventions using health behaviour theories. Click here for file
