# Supplementary material for: A review of health behaviour theories: how useful are these for developing interventions to promote long-term medication adherence for TB and HIV/AIDS?
Source: BMC Public Health. 2007 Jun 11;7:104. doi: 10.1186/1471-2458-7-104 (PMC1925084; doi:10.1186/1471-2458-7-104)
Supplement: Additional file 1 — Examples of interventions using health behaviour theories. [file 1471-2458-7-104-S1.DOC]

Table 3: Examples of interventions using health behaviour theories

| Theory | **Citation** | **Setting** | **Study method** | **Aim** | **Intervention/s** | **Results** |
| --- | --- | --- | --- | --- | --- | --- |
| HBM | 89 | Singapore | Randomised controlled trial (RCT) | Increase medication adherence of geriatric pharmacy clients | Pharmacist consultation to minimise therapeutic duplication; simplify regimen; improve effectiveness of regimen and decrease adverse drug reactions and cost (decrease barriers); and improve medication knowledge, clinical status and perception (increase perceived benefits). Specific counselling and adherence boosting strategies for nonadherent patients. | Increased adherence |
| PMT | 90 | United States | Experimental before-after study with random assignment | To increase adherence and clinical outcomes for asthma patients | Theoretically focussed audiotape addressing vulnerability, severity, self-efficacy and response efficacy, or standard educational booklet or both. | Booklet alone increased adherence at 3 and 6 months; combination increased adherence at 6 months. |
| SCT | 91 | Spain | RCT | To improve long-term adherence to highly active antiretroviral therapy | Received explanation of reasons for starting treatment and need for adherence; doubts discussed and resolved; dosage schedules developed collaboratively; and participants were taught to manage their medication. During follow up visits adherence was verbally reinforced and strategies developed to solve emerging problems. | Weakly significant improvement in adherence at week 48. |
| TPB | 92 | UK | RCT | To improve the dietary habits of hospital workers | Establishing participants’ salient beliefs, reinforcing correct ones and refuting incorrect ones. | Improved attitudes, limited effect on fat consumption |
| Self regulatory | 93 | The Netherlands | RCT | To increase adherence to treatment of essential hypertension | Discussing emotional or cognitive representations that might hinder adherence; or discussing plans for adherence action | No difference |
| Stages of change (SOC) | 94 | UK | Cluster RCT | To prevent and end smoking in school-children | Students completed a computerised questionnaire and received feedback on their ‘stage’ and how it had changed since the last session. Questionnaires were interspersed with recordings, relevant to the stage of change of young people talking about smoking. One hour lesson on stages of change as control. | No difference |
| IMB | 17 | United States | RCT | To promote adherence to antiretroviral therapy | Medication manager provided tailored adherence support over time, identifying and addressing information, motivation and skills. | Lower rate of virologic failure on or after 4 months. |
